# Eating disorders in relation to alcohol addiction—A study of drunkorexia in young adults in Poland

**DOI:** 10.3389/fpubh.2025.1629206

**Published:** 2025-07-18

**Authors:** Marta Giezek, Marek Landowski, Paulina Zabielska, Andriej Szpakow, Beata Karakiewicz

**Affiliations:** ^1^Subdepartment of Social Medicine and Public Health, Department of Social Medicine, Pomeranian Medical University in Szczecin, Szczecin, Poland; ^2^Independent Research and Biostatistics Laboratory, Department of Social Medicine, Pomeranian Medical University in Szczecin, Szczecin, Poland; ^3^Department of Nursing, International Academy of Applied Sciences in Łomża, Łomża, Poland

**Keywords:** addictions, alcoholism, eating disorders, drunkorexia, Poland

## Abstract

**Introduction:**

The aim of the study was to determine the frequency of eating disorders occurring in combination with alcohol dependence in young adults in Poland.

**Material and methods:**

The study was conducted in Poland between 1st July 2023 and 31st July 2024. The diagnostic survey method was applied. The following tests were used to identify problems: AUDIT (Alcohol Use Disorders Identification Test) for assessing alcohol consumption, drinking behaviors, and alcohol-related problems, and EAT 26 (Eating Attitudes Test) for the psychometric assessment of eating disorders.

**Results:**

Statistical analysis of the average AUDIT and EAT-26 survey scores according to gender and age (under 25 years and from 25 to 35 years) revealed a statistically significant difference in results between the male and female groups. The average AUDIT survey score for women was lower than for men and amounted to 4.42 and 6.77, respectively. This might indicate that men engage in alcohol consumption more frequently than women. However, for the EAT-26 survey, the situation was the opposite, an average score was higher for women than for men and amounted to 10.7 and 6.71, respectively. In this case, this result could indicate that women have a greater tendency to eating disorders or that women more frequently use weight loss diets than men. The study group included young adults with an AUDIT score greater than or equal to 15, which indicates the probability of alcohol addiction. As many as 80.95% of women and 44% of men from this group might also suffer from eating disorders.

**Conclusions:**

The article draws attention to a relatively new, especially in Poland, public health problem which requires the introduction of appropriate preventive measures, particularly in the area of raising awareness of the negative effects of treating alcohol as a regulator of emotions and in the area of nutritional education. The research results and the description of the problem are a useful contribution to the practice of doctors and nurses in primary health care allowing them to differentiate drunkorexia from alcoholism and eating disorders and introduce early therapeutic procedures in order to prevent serious health and social consequences.

## Introduction

The study of drunkorexia in young adults in Poland was undertaken as a result of their ever-increasing alcohol consumption and their drive for thinness. Although behaviors related to alcohol consumption and eating behaviors in this age group have been widely studied, they have only been analyzed as separate phenomena. Drunkorexia, however, is a very complex and serious health problem belonging to behavioral disorders and is characterized by limiting food consumption in order to allow for larger alcohol intake without gaining body weight.

**Eating disorders** are abnormal behaviors in the area of eating habits which lead to significant disturbances in body weight. They constitute a significant health problem predominantly affecting girls and young women. These disorders cause many negative consequences, both psychological, social and health-related. The complex etiology of eating disorders requires an interdisciplinary approach, including lifestyle modification, especially in relation to eating behaviors, psychological support or pharmacological treatment (1, 2).

**Alcohol addiction** is a set of somatic, behavioral and cognitive symptoms associated with excessive, compulsive consumption of alcoholic beverages without controlling the frequency and amount of consumption, which consequently leads to physical and mental debilitation of the body (3).

**Drunkorexia** is a combination of eating disorders with alcohol addiction, which is also described as alcoholic anorexia, alcoholic bulimia or alcoholic anorexia (4). Drunkorexia is a behavioral disorder characterized by restriction of food intake in order to allow for the consumption of larger amounts of alcohol without gaining weight. The term drunkorexia first appeared in 2008, but it has not yet been formally classified as a complex disorder. The first scientific research on drunkorexia was conducted at the University of Missouri by Victoria Osborne in 2011 (5, 6). Drunkorexia is not a separate and official medical term in the ICD-11 classification (International Classification of Diseases), which causes problems with its diagnosis as it is confused with anorexia (7, 8). The main factors differentiating drunkorexia from anorexia are presented in Table 1.

**Table 1:** Differentiation between drunkorexia and anorexia.

**Drunkorexia**	**Anorexia**
**Aim**	
Giving up food due to the desire to drink larger amounts of alcohol without the resultant weight gain	Weight loss due to self-perception as an obese and unattractive person.
**Frequency**	
Only in situations where alcohol consumption is intended	Persistent, constant behavior, with no breaks in the improper diet.

Source: Authors' own study based on (4, 6, 9).

The use of term “alcoholic bulimia” for drunkorexia results from the mechanism and possible course of the disease. Alcohol, by stimulating appetite and not providing a feeling of satiety, causes attacks of compulsive overeating, which encourages taking actions aimed at disposing of excess food in order to maintain a slim figure and a desired body weight (10, 11).

The predictors of drunkorexia are mainly age (young adults) and gender (women more often than men). This disorder is relatively new, and the number of publications on the topic is not extensive. However, the studies conducted so far indicate that first-year female students are particularly at risk due to their entering a new peer group, lack of parental control, and a change in the style of studying and free time activities (12, 13).

The causes of drunkorexia are defined in three dimensions: individual, situational and biological. Individual predispositions of drunkorexia are mainly personality factors, including: low self-esteem, negative body image, the desire to be perfect, as well as experienced traumas or persistent emotional pain (14). In the situational dimension, the most important role is played by social factors, which include the cult of a slim body and the attitude toward alcohol consumption, i.e. acceptance of drinking alcohol during social gatherings and treating alcohol as an antidote to stress (6). Biological factors involve the individual's tendency to eating disorders and alcohol abuse (4).

When diagnosing drunkorexia, the diagnosis of alcohol dependence is of particular importance. During the interview, it should be determined which disorder appeared first – whether the eating disorder is a result of the phase of alcoholism in which the alcohol-dependent person loses interest in eating, or whether the eating disorder is a primary problem, to which the problem of alcohol abuse has been added (4, 15).

Alcoholism develops in four successive phases, to which eating disorders can be assigned, in contrast to drunkorexia, which is characterized by a self-imposed, intentional and restrictive calorie restriction prior to alcohol consumption, as shown in Table 2.

**Table 2:** The relationship between drunkorexia and alcohol addiction.

**Alcohol addiction**			**Drunkorexia**
**Phase of alcoholism**	**Control of alcohol consumption**	**Eating disorders**	**Weight gain control**
Pre-alcoholic phase	- Control over drinking, - Alcohol consumption does not differ much from the socially accepted norm, - Drinking is social in nature, - Drinking is enjoyable and brings various benefits, including relaxation and higher self-esteem.	No disturbances in the regulation of food intake	- Occasional weight checks - Calorie restrictions before social events
Prodromal (warning) phase	- Partial control over drinking, - Beginning of regular drinking - Experiencing the “magical” effect of alcohol on well-being, - Increased focus on alcohol, looking for opportunities to drink, initiating drinking, hiding drinking habits from others, drinking alcohol alone.	First abnormalities in nutritional behavior	- Increased frequency of body weight checks - Focus on the amount of meals consumed and their caloric value
Critical phase	- Loss of control over drinking, - Drinking alcohol to the point of intoxication. - Denying problems related to drinking, - neglecting work, family and losing other interests, - Concentrating life on alcohol, - Accumulating alcohol supplies, - The need to constantly replenish the alcohol concentration in the blood.	- Numerous nutritional abnormalities - Irregular meals, - Low nutritional value	- Very frequent weight checks, - Hunger attacks, - Compulsive overeating, - Self-induced vomiting, - Taking laxatives, - Living in fear of gaining weight.
Chronic phase	- Complete lack of control over drinking - Morning drinking, - Getting drunk alone, - Decreased tolerance for alcohol, - reaching for non -Beverage alcohol, - Thefts in order to obtain alcohol - Alcoholic psychosis, alcoholic epilepsy, - Somatic diseases such as: alcoholic liver cirrhosis, alcoholic polyneuropathy, alcoholic dementia, extreme debilitation of the body.	- Constant loss of interest in food	- Drinking alcohol on an empty stomach, - Starving yourself, - Replacing food energy intake with calories from alcohol consumption.

Source: Authors' own study based on (4, 16–18).

**Figure 1:**
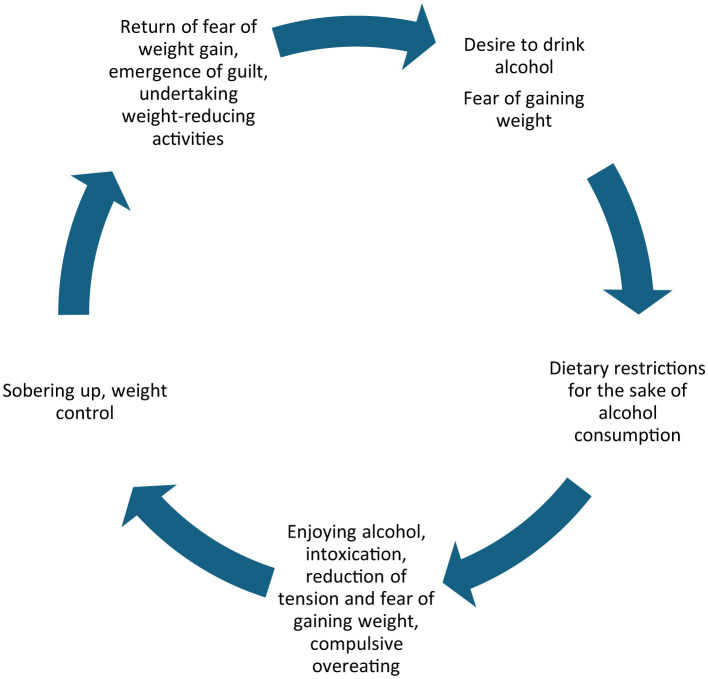
Drunkorexic cycle—authors' own study based on (4, 6, 19, 20).

The analysis of the literature enables formulation of a mechanism which may be described as the drunkorexic cycle. Awareness of the calorific value of alcohol and fear of gaining weight cause a reduction in the supply of meals—initially occasional when drinking alcohol socially. Then, along with the growing pleasure of drinking alcohol, the fear of gaining weight increases, which causes further dietary restrictions. This is followed by drinking until intoxication, which in turn reduces tension, eliminates anxiety, and brings a sense of relaxation. In the sobering up phase, the body weight and fear of gaining weight are analyzed again, but the strong desire to consume alcohol causes the introduction of further action aimed at the reduction of the consumed calories (Figure 1).

Deliberate giving up food in favor of alcohol consumption so as to prevent weight gain and to maintain the desired weight, combined with fasting, self-induced vomiting and taking laxatives lead to extreme body debilitation and the development of alcoholism.

The article constitutes a significant contribution to the currently available, rather sparse literature on the problem of drunkorexia. It describes the situation in a country where 84% of the adult population engage in alcohol consumption. In Poland, almost all adult men (90%) and most adult women (79%) drink alcohol, as well as more than half (55.6%) of young people (15–19 years old). Over the last 20 years, the quantity of alcohol production has increased. The rate of pure alcohol consumption per capita for 15+ has been 11.89 l, which means that Polish society is on the verge of a degrading level in this respect.^1^

## Aim

The aim of the study was to determine the frequency of occurrence of eating disorders combined with alcohol addiction in young adults in Poland.

## Methods

The type of research presented in this article is a descriptive cross-sectional study. The study was conducted in Poland between 1st July 2023 and 31st July 2024. The only inclusion criterion was the age of the respondents within the range of 18–35 years. The study was conducted online, using the Google Forms survey creator. A link and QR code were provided to complete the survey titled: “Eating disorders and alcohol addiction in young adults - a study of the phenomenon of drunkorexia.” Participation in the survey was anonymous, voluntary, and free of charge. The study design obtained the approval from the Bioethics Committee of the Pomeranian Medical University KB.006.82.2023 on 30th June 2023.

The study was carried out using the diagnostic survey method. The following tools were used in order to identify problems:

1. **The AUDIT** (Alcohol Use Disorders Identification Test) - a 10-question research tool developed by the World Health Organization (WHO) to assess alcohol consumption, drinking behaviors, and alcohol-related problems. It consists of 10 questions which refer to various aspects of alcohol consumption. Adaptation and validation of the AUDIT test to Polish conditions (21).
2. **EAT 26** (Eating Attitudes Test) is used for psychometric assessment of eating disorders. A screening tool for assessing symptoms associated with eating disorders. It consists of 26 items divided into 3 subgroups: dieting, bulimia and eating control, and oral control. This allows for the assessment of attitude and behavior in three symptom areas which are closely related to eating disorders. The final score is the sum of all groups. A score above 20 points is considered positive. Polish adaptation: Rogoza et al. (21).
3. **Personal data questionnaire** – consisting of 13 questions, 8 of which concern sociodemographic characteristics and 5 cover the issue of alcohol use and eating disorders.

### Participants

The survey was completed by 1,017 respondents. Due to the lack of complete data, 3 surveys were excluded from the analysis. Additionally, in view of the fact that the study covered people up to 35 years of age, another 62 surveys were rejected, where the respondents were over 35 years of age. A total of 952 surveys remained eligible for analysis.

The mean age and standard deviation of the group of 952 respondents was 22.27 ± 4.1 years. The group comprised 72.06% women, 27% men and 0.94% of people of the “other/unspecified” gender. The mean age of women and men was about 22 years, while for the group of people of the “other/unspecified” gender the mean age was 19.89 years. As many as 80.46% of the respondents were aged 18–24, with the mean age of 20.56 years, the remaining 19.54% were aged 25-35, with the mean age of 29.33 years.

The majority of respondents live in the city (71.85%). Being in a relationship is declared by 50.21% of respondents, the remaining 49.79% describe themselves as single. The largest group of respondents, 75.32%, are students/pupils. The majority of respondents have secondary education (70.48%).

Table 3 presents the sociodemographic characteristics of the respondents and the mean age with standard deviation, median age with interquartile deviation, and age range.

**Table 3:** Sociodemographic characteristics of respondents.

**Group of respondents**	***N***	**Age (Mean ± SD)**	**Age (Median ± QD)**	**Age (min–max)**
All respondents	952	22.27 ± 4.1	21 ± 2	18–35
**Gender**				
Women	686 (72.06%)	22.14 ± 4.18	21 ± 2	18–35
Men	257 (27%)	22.7 ± 3.91	21 ± 2	18–35
Other/unspecified	9 (0.94%)	19.89 ± 1.05	20 ± 1	18–21
**Age**				
< 25 years old	766 (80.46%)	20.56 ± 1.63	20 ± 1.5	18–24
25–35 years old	186 (19.54%)	29.33 ± 3.62	29 ± 3.5	25–35
**Domicile**				
City	684 (71.85%)	22.5 ± 4.26	21 ± 2	18–35
Village	268 (28.15%)	21.69 ± 3.6	21 ± 2	18–35
**Marital status**				
In a relationship	478 (50.21%)	22.99 ± 4.52	22 ± 2	18–35
Single	474 (49.79%)	21.55 ± 3.48	20 ± 1.5	18–35
**Education**				
Higher	263 (27.63%)	26.16 ± 4.5	25 ± 3.5	21–35
Secondary	671 (70.48%)	20.8 ± 2.77	20 ± 1	18–35
Vocational	10 (1.05%)	21.4 ± 2.07	21 ± 0	19–25
Primary	8 (0.84%)	19.63 ± 1.3	19 ± 0.75	18–22
**Employment**				
Students	717 (75.32%)	20.84 ± 2.65	20 ± 1.5	18–35
White-collar worker	133 (13.97%)	28.22 ± 4.66	28 ± 4.5	20–35
Manual worker	96 (10.08%)	24.58 ± 3.64	24 ± 2	19–35
Unemployed	6 (0.63%)	25.17 ± 4.22	25 ± 1.5	20–32

### Statistical analysis

Quantitative data were presented as mean, standard deviation, median and interquartile deviation, as well as percentages. The Shapiro-Wilk test was used to examine the normality of the distribution, while the chi-squared test, Kruskal-Wallis rank sum test and U Mann-Whitney test were used to analyse differences between distributions. The significance level of 0.05 was assumed for all tests. Cronbach's alpha was used to measure the reliability of rating scale questions. The Cronbach's alpha coefficient for the AUDIT survey was 0.8, while for the EAT-26 survey this coefficient had the value of 0.88, therefore the overall reliability of the questionnaires was achieved.

## Results

The mean value of the AUDIT survey was 5.08. The minimum and maximum values which could be obtained in the survey were 0 and 30. Analyzing the mean values in four ranges of AUDIT score, the largest number of respondents were classified into the second range, indicating low alcohol consumption, i.e. 654 respondents (68.7%). The mean value of points in this case was 3.59. Respondents with zero points, defined as abstainers, who had never had problems with alcohol constituted 9.24% of the group. People with a score of 8–14, which suggested hazardous or harmful alcohol consumption, constituted 17.12% of all respondents—the mean in this group was 9.7. The last group with the highest AUDIT score, 15 or more, which indicated the probability of alcohol dependence, consisted of 47 respondents (4.94%), with a mean of scores of 19.21.

As regards the EAT-26 survey, the mean value for 952 respondents was 9.65 ± 10.34. A score of 20 or more, which for this survey indicates a possible eating disorder, was received by 130 people (13.66% of the participants). The mean in this case was 31.48. The remaining 86.34% of respondents (822 people), had scores below 20, with a mean of 6.2. The EAT-26 survey questions according to Garner (22) were divided into three subscales: Dieting −13 questions, Bulimia & Food Preoccupation −6 questions, Oral Control −7 questions. In the individual groups, the mean values was 6.58, 1.39 and 1.68, respectively. The results of the AUDIT and EAT-26 questionnaires are presented in Table 4.

**Table 4:** Results for the AUDIT and EAT-26 questionnaires (in total and divided into dieting, bulimia & food preoccupation, and oral control).

**Questionnaire**	***N***	**Score**	**Score**	**Score**
		**Mean** ± **SD**	**Median** ± **QD**	**Min–Max**
**AUDIT**	952	5.08 ± 4.62	4 ± 2.5	0–30
Score is 0	88 (9.24%)	0	0	0
Score from 1 to 7	654 (68.7%)	3.59 ± 1.91	3 ± 1.5	1–7
Score from 8 to 14	163 (17.12%)	9.7 ± 1.79	9 ± 1.5	8–14
Score of 15 or more	47 (4.94%)	19.21 ± 4.25	18 ± 4	15–30
**EAT-26–26 Qs**	952	9.65 ± 10.34	6 ± 5	0–56
Score from 0 to 19	822 (86.34%)	6.2 ± 4.96	5 ± 3.5	0–19
Score of 20 or more	130 (13.66%)	31.48 ± 8.65	30 ± 6.5	20–56
**EAT-26**				
Dieting – 13 Qs	952	6.58 ± 6.89	4 ± 4	0–33
Bulimia & food preoccu. – 6 Qs	952	1.39 ± 2.77	0 ± 0.5	0–15
Oral control−7 Qs	952	1.68 ± 2.58	0 ± 1.5	0–17

Part of the EAT-26 questionnaire called “behavioral questions” indicates a possible risk of an eating disorder (22). According to Garner (22), meeting at least one of the three criteria should prompt the individual to consult a specialist in the treatment of eating disorders. The criteria mentioned by Garner are:

- EAT-26 score at or above 20,
- BMI value < 18.5, i.e. at a level symptomatic of underweight,
- the possibility of an eating disorder suggested by the behavioral questions or significant weight loss in the last 6 months.

Respondents who were underweight (BMI < 18.5) were determined using the following WHO-defined baseline BMI (kg/m^2^) categories: (severe thinness: BMI < 16, moderate thinness: BMI in 16–16.99, mild thinness: BMI in 17–18.49) (22, 23).

A total of 396 respondents (41.6%) met at least one of the above criteria, and 29 of them (3.05% of all respondents) indicated the probability of alcohol addiction according to the AUDIT test (AUDIT score was 15 or more). The average AUDIT score in this group was 19.48, the average age of respondents was 22.9 years. Conversely, 80 out of the indicated 396 respondents at risk of an eating disorder (8.4% of all respondents) had an AUDIT score 8–14 points, suggesting hazardous or harmful alcohol consumption (average score of 9.93). The average age with standard deviation in this group was 22.7 ± 4.48 years. There were 247 respondents with a possible eating disorder who consumed alcohol with low health risk (AUDIT score from 1 to 7), which constituted 25.95% of all respondents. The average AUDIT score in this case was 3.71, and the average age of respondents was 21.89 years. There were 40 respondents indicating the possibility of an eating disorder who were abstinent (AUDIT score = 0). This value constituted 4.2% of those analyzed with an average age of 21.33 years.

The group of 29 respondents with the indicated possibility of eating disorders and alcohol addiction consisted of 17 women, 11 men and 1 person of other/unspecified sex (average age of 22.17, 24.27 and 20 years, respectively). In this group, 19 people lived in the city (average age 24.21), 10 people lived in a village (average age 20.4); 18 people were in a relationship (average age 24), and 11 people were single (average age 21.09); 8 of them had higher education, 20 had secondary education, and 1 person has basic education (average ages were 26.88, 21.23 and 22 years, respectively).

Table 5 presents the results for respondents with a probability of eating disorders and alcohol dependence.

**Table 5:** Results for respondents with possible eating disorders and alcohol dependence.

**Questionnaire/Group of respondents**	***N***	**AUDIT score**	**Audit score**	**Age**	**Age**
		**Mean** ± **SD**	**Min–Max**	**(Mean** ± **SD)**	**Min–Max**
**AUDIT and possibility of eating disorders (** ^*^ **)**	396	5.75 ± 5.32	0–30	22.07 ± 3.99	18–35
AUDIT score is 0	40	0	0	21.33 ± 4	18–34
AUDIT score from 1 to 7	247	3.71 ± 2.04	1–7	21.89 ± 3.73	18–35
AUDIT score from 8 to 14	80	9.93 ± 2	8–14	22.7 ± 4.48	18–35
AUDIT score of 15 or more	29	19.48 ± 4.61	15–30	22.9 ± 4.57	18–32
AUDIT score ≥ 15 and (^*^)	
**Gender**	
Women	17	18.47 ± 3.59	15–27	22.17 ± 4.5	18–32
Men	11	21.45 ± 5.56	15–30	24.27 ± 4.71	19–32
Other/unspecified	1	15	15	20	20
**Domicile**	
City	19	20 ± 5.3	15–30	24.21 ± 4.8	18–32
Village	10	18.5 ± 2.88	15–24	20.4 ± 2.88	18–27
**Marital status**	
In a relationship	18	18.11 ± 4.34	15–28	24 ± 5.09	18–32
Single	11	21.73 ± 4.31	16–30	21.09 ± 2.95	18–27
**Education**	
Higher	8	20.25 ± 5.65	15–28	26.88 ± 3.83	22–32
Secondary	20	19.2 ± 4.38	15–30	21.35 ± 4.02	18–31
Basic	1	19	-	22	-

(^*^) EAT-26 score ≥ 20 or BMI < 18.5 or behavioral questions ≥ 1 or significant weight loss.

The statistical tests of the average value of AUDIT and EAT-26 survey points by gender and age (under 25 years and from 25 to 35 years) showed a statistically significant difference in results between the group of women and men. The average value of AUDIT survey points for women was lower than for men and amounted to 4.42 and 6.77, respectively. This might indicate that men drink alcohol more frequently than women. However, for the EAT-26 survey, the situation was the opposite, with women presenting a higher average value of points than men, which was 10.7 and 6.71, respectively. In this case, this result might indicate women's greater tendency to eating disorders, or the fact that women more frequently use weight loss diets than men.

There was no statistically significant difference between the results of people declaring “other/unspecified” gender and “female” or “male” gender, most probably due to a very small number of respondents declaring this gender. In the “other/unspecified” gender group the average AUDIT survey score was 6.56 while the average EAT-26 survey score amounted to 13.89. Note that there were 9 respondents who identified their gender as “other/unspecified” in the group of respondents, compared to several hundred respondents who identified their gender as “women” or “men”. This fact may weaken the analytical sensitivity of detecting differences. This remark applies only to tests involving respondents who identified their gender as “other/unspecified.”

Statistical tests did not show a statistically significant difference between the mean values of the responses of people under 25 and those aged 25–35. In this case, the mean value of the AUDIT and EAT-26 survey scores for people under 25 was 4.95 and 9.97, respectively, while for people aged 25–35 these indicators were 5.61 and 8.35. The mean AUDIT survey score was higher among older people, while for the EAT-26 survey the situation was the opposite, with a higher mean score obtained by people under 25. As mentioned earlier, these differences were not statistically significant. The results of the mean survey scores according to gender and age are presented in Table 6.

**Table 6:** Results of the AUDIT and EAT-26 surveys and the Kruskal-Wallis rank sum test and U Mann-Whitney test for selected respondent groups.

**Questionnaire/Group of respondents**	***N***	**AUDIT score**	**Statistical test result**					
		**Mean** ± **SD**						
**AUDIT**	
All resp.	952	5.08 ± 4.62						
**Gender**			H-stat	*Df*	*p*	A vs. B	A vs. C	B vs. C
A: Women	686	4.42 ± 3.91	38.7	2	< 0.01	< 0.01	0.48	1.00
B: Men	257	6.77 ± 5.79						
C: Other/unspecified	9	6.56 ± 4.93						
**Age**			Z-stat	*P*				
A: < 25 yo	766	4.95 ± 4.46	1.21	0.22				
B: 25–35 yo	186	5.61 ± 5.21						
**EAT-26**								
All resp.	952	9.65 ± 10.33						
**Gender**			H-stat	*df*	*p*	A vs. B	A vs. C	B vs. C
A: Women	686	10.7 ± 11.32	10.21	2	< 0.01	< 0.01	1.00	0.74
B: Men	257	6.71 ± 5.76						
C: Other/unspecified	9	13.89 ± 16.64						
**Age**			Z-stat	*p*				
A: < 25 yo	766	9.97 ± 10.62	−1.95	< 0.051				
B: 25–35 yo	186	8.35 ± 8.99						

The group of 9 people declaring “other/unspecified” gender was excluded from the further part of the chi-squared tests, as the number of respondents was too small. Chi-squared tests were performed for respondents declaring “women” and “men” gender.

Considering the AUDIT and EAT-26 survey scores, the chi-squared test showed a statistically significant dependence of the survey scores on the respondents' gender. The value of AUDIT points was dependent on gender (*p* < 0.001). The percentage of women was lower than the percentage of men in the case of a high number of points. The AUDIT survey score above 7 points, which indicates dangerous, harmful alcohol consumption or the probability of alcohol addiction, was obtained by 35.41% of men and 16.91% of women. The lowest value of 0, pointing to a teetotaller who has never had problems with alcohol, was obtained by 9.91% of women and 7.78% of men.

For the EAT-26 survey, the dependence was reversed, with more women obtaining higher scores than men. The EAT-26 survey score above 19, which indicates possible eating disorders, was obtained by 17.49% of women compared to 2.72% of men. The answers to the “behavioral questions” were also statistically significantly dependent on the gender of the respondents. Women (31.2%) more frequently provided affirmative answers to these questions than men (17.51%). No statistically significant relationship was observed in the response to the question concerning weight loss in the recent period. The percentages of affirmative answers to this question provided by women and men were comparable and amounted to 9.77% and 9.34%, respectively. The BMI value of the respondents was dependent on their gender. A higher percentage of women (9.04%) than men (2.72%) had a BMI < 18.5.

The analysis of the possibility of eating disorders in the surveyed individuals according to the indicators provided by Garner (22), i.e. EAT-26 score ≥ 20 or BMI < 18.5 or behavioral questions ≥ 1 or significant weight loss also demonstrated a statistically significant relationship between the risk of eating disorders and gender (*p* < 0.001). The possibility of eating disorders was indicated in 46.5% of women and in 26.85% of men.

The next stage of the study was to select women and men with an EAT-26 questionnaire indicator with a value suggesting eating disorders or BMI < 18.5 and to analyse the percentage of women and men with an AUDIT questionnaire score in four cases: score = 0, 1-7, 8-14 and ≥ 15. The chi-squared test showed a statistically significant correlation between the AUDIT questionnaire scores and the respondents' gender in all four cases. Of the respondents with an AUDIT score = 0, denoting a person who has never had problems with alcohol, 51.47% of women showed a possibility of eating disorders [according to Garner (22)], while for men this value was 25%. Of the respondents classified as those with a low risk of alcohol consumption (AUDIT score from 1–7), 41.43% of women showed a possibility of eating disorders, compared to 22.6% of men. The next group with AUDIT score 8-14 (hazardous or harmful alcohol consumption), comprised 62.11% of women and 30.3% of men presenting the risk of eating disorders. In the last group of respondents with AUDIT score ≥ 15, which indicates the probability of alcohol dependence, 80.95% of women and 44% of men might also have eating disorders (according to Garner's 23 indexes). The described results for AUDIT and EAT-26 questionnaires depending on the gender of the respondents and the results of the performed chi-squared tests are presented in Table 7.

**Table 7:** AUDIT and EAT-26 survey results according to respondents' gender.

**Questionnaire/Group of respondents**	**Women**	**Men**	**Chi-sq**	***df***	***P***
**AUDIT**					
Score = 0	68 (9.91%)	20 (7.78%)	40.56	3	< 0.001
Score from 1 to 7	502 (73.18%)	146 (56.81%)			
Score from 8 to 14	95 (13.85%)	66 (25.68%)			
Score of 15 or more	21 (3.06%)	25 (9.73%)			
**EAT 26**					
Score below 20	566 (82.17%)	250 (97.28%)	34.99	1	< 0.001
Score at or above 20	120 (17.49%)	7 (2.72%)			
**EAT-26 behavioral questions**					
Yes	214 (31.2%)	45 (17.51%)	17.58	1	< 0.001
No	472 (68.8%)	212 (82.49%)			
**EAT-26 significant lost weight**					
Yes	67 (9.77%)	24 (9.34%)	0.039	1	0.84
No	619 (90.23%)	233 (90.66%)			
**BMI**<**18.5**					
Yes	62 (9.04%)	7 (2.72%)	10.99	1	< 0.001
No	624 (90.96%)	250 (97.28%)			
**Possibility of eating disorders (** ^*^ **)**					
Yes	319 (46.5%)	69 (26.85%)	29.82	1	< 0.001
No	367 (53.5%)	188 (73.15%)			
**AUDIT score** = 0 and (^*^)					
Yes	35 (51.47%)	5 (25%)	4.37	1	< 0.04
No	33 (48.53%)	15 (75%)			
**AUDIT score 1–7 and (** ^*^ **)**					
Yes	208 (41.43%)	33 (22.6%)	17.17	1	< 0.001
No	294 (58.57%)	113 (77.4%)			
**AUDIT score 8–14 and (** ^*^ **)**					
Yes	59 (62.11%)	20 (30.3%)	15.76	1	< 0.001
No	36 (37.89%)	46 (69.7%)			
**AUDIT score** ≥ 15 and (^*^), i.e. possibility of eating disorders and alcohol dependent					
Yes	17 (80.95%)	11 (44%)	5.08 (^**^)	1	< 0.05
No	4 (19.05%)	14 (56%)			

(^*^) EAT-26 score ≥ 20 or BMI < 18.5 or behavioral questions ≥ 1 or significant weight loss.

(^**^) Yates correction.

## Discussion

Excessive alcohol consumption is a significant public health problem. The study results are consistent with the data from the second edition of the “Alcohol in Poland” report (2023), which shows that 73% of alcohol consumers drink alcohol in moderation (up to 6 liters of pure alcohol per year), 27% drink in a risky and harmful manner (over 6 liters of pure alcohol per year), including 11.6% who abuse alcohol (24). In comparison with the results of statistical surveys (2019), there is a noticeable increase in the percentage of people drinking excessively and harmfully by 8 percentage points.

Changes occurred both in the group drinking the least—up to 1.2 l per year (decreased from 46.7% to 35.9%) and in the groups drinking hazardously (increased from 11.3% to 15.3%) and excessively and harmfully (increased from 7.3% to 11.6%) (25).

Considerable differences in the distribution of pure alcohol consumption can be seen in the division by gender. A significantly higher percentage of women drink moderately, up to 1.2 liters of pure alcohol per year (56.8%), and a significantly higher percentage of men drink in a risky, harmful and abusive manner (43.5% in total). As regards the distribution of alcohol consumption in individual age groups, the highest number of moderate drinkers (up to 6 liters of pure alcohol per year) is among the youngest (18–34 years old) – almost 74%, and the oldest (65 years old +) – 79.7% (24, 25).

Apart from alcohol abuse, eating disorders are also common in Poland. It is estimated that in the adult population, the incidence of anorexia is 0.5–1% in women and 0.05–0.1% in men (26), while the incidence of bulimia is estimated at 1.3% in the adult population (27). In addition to these well-known disorders, there is an increasing discussion of so-called non-specific eating disorders, which include orthorexia, bigorexia and drunkorexia. Individuals suffering from eating disorders more often admit to increased alcohol consumption (28). Approximately 40% of American students report drinking until intoxication at least once in two weeks (11, 14). Studies have shown that the largest amounts of alcohol are consumed by students in their first year of studies, which makes them the largest group at risk. Along with excessive alcohol consumption, students commonly suffer from eating disorders. Approximately 60% of female students report chronic use of weight loss diets and binge eating. Moreover, most of the surveyed female students admit to using various methods aimed at controlling their body weight (11). Approximately 55% of the surveyed American and French students showed behaviors related to drunkorexia (29). A study conducted on an Italian sample showed that in 32.2% of participants limiting calorie intake before drinking was more common among people who regularly consumed alcohol, compared to people who drank occasionally (30). The few scientific reports on drunkorexia from other countries prompted the study to be conducted in Poland. A sample of young adults was selected as the most predisposed group (4, 31). The obtained results of the study are consistent with those described by Eisenberg and Fitz (32), which concluded that women who consumed larger amounts of alcohol were more likely to develop drunkorexia than women who consumed alcohol in smaller amounts. Both research processes also concluded that women were more concerned about gaining weight than men, and therefore women who drank large amounts of alcohol and were strongly motivated to control their body weight were most likely to develop drunkorexia. The study conducted in a group of young Polish adults showed that 23.63% skipped a meal or restricted their caloric intake in favor of alcohol consumption. In this group, as many as 21.33% resorted to this behavior very often and often, and almost 5% always. The study results are also consistent with those described by Oswald et al. (33), which show that about 23% of people declared calorie restriction on days when they planned to drink alcohol, so that the increased calorie intake from alcohol would not cause weight gain.

An interesting phenomenon was observed in Poland during the COVID 19 pandemic. During the two pandemic years, the total consumption of pure alcohol per capita in Poland decreased compared to 2019: in 2020 it amounted to 9.62 liters per capita, in 2021−9.7 liters per capita, which is a decrease of 0.16 liters and 0.08 liters, respectively (34). Conversely, the Global Drug Survey (GDS) conducted in May and June 2020 on 55 thousand respondents from 12 countries (Australia, Austria, Brazil, France, Germany, Ireland, the Netherlands, New Zealand, Switzerland, the United Kingdom and the USA) indicated that 36% of consumers increased their alcohol consumption in the first year of the pandemic, 22% consumed smaller amounts, and 42% remained at the same level of consumption. An even larger percentage declared an increase in the frequency of drinking (46%). The GDS report also indicated that during the pandemic, as many as 30% of respondents admitted that they started drinking earlier in the day than before 2020, and in the case of women, this percentage was even higher and amounted to 32%. The main reasons for increased and more frequent alcohol drinking according to the GDS report were primarily related to the mental state of the respondents, stress, a sense of loneliness, as well as the fact that when working or studying remotely, they were subject to less social control and had more free time.^2^

## Limitations

The limitation of the study was the focus on the age group, namely young adults, which prevented the establishment of the frequency of drunkorexia in mature adults and older people. The research tools used did not make it possible to determine whether the reduction of caloric intake prior to alcohol consumption was caused by fear of weight gain or by the desire to achieve faster effects of alcohol. The lack of previous studies precluded comparison of the obtained results with the determination of suspicion of drunkorexia, e.g. in the period before the COVID 19 pandemic.

## Conclusions

The article confirms that there is a problem of drunkorexia in young adults in Poland. However, this study should be treated as an introduction to further exploration of the subject by expanding the research sample to include mature adults and older people. Ambiguous diagnostic criteria for drunkorexia make it difficult to recognize and differentiate it from other eating disorders and alcohol addiction. Currently available diagnostic categories allow for separate recognition of alcoholism and/or eating disorder. The development of a scale for examining drunkorexia and distinguishing it as a disease entity would allow for early diagnosis and introduction of therapy. The article draws attention to a relatively new public health problem, especially in Poland, which requires introduction of appropriate preventive measures - particularly in the area of raising awareness of the negative effects of treating alcohol as a regulator of emotions and in the area of nutritional education. The drunkorexic cycle described in the article, the description of the problem and the results of the study are a useful contribution to the practice of doctors and nurses in primary health care, allowing them to differentiate drunkorexia from alcoholism and eating disorders and introduce early therapeutic procedures in order to prevent serious consequences which particularly concern young women.

## Data Availability

The raw data supporting the conclusions of this article will be made available by the authors, without undue reservation.

## Footnotes

^1^Available online at: https://www.parpa.pl/images/Adaptacja_i_walidacja_testu_AUDIT_do_warunk%C3%B3w_polskich.pdf (Accessed December 27, 2024).

^2^Available online at: https://www.globaldrugsurvey.com/gds-covid-19-special-edition-key-findings-report/ (Accessed December 31, 2024).
